# Long‐Term Follow‐Up of Nonlethal Raine Syndrome in an Omani Child: A Case Report

**DOI:** 10.1155/crpe/5318301

**Published:** 2026-11-13

**Authors:** Hanan Al Azkawi, Shaima Al Madhani, Nagla Nassor, Wafaa Abdelrahman, Omer Ahmed, Mouza ALYahyaei

**Affiliations:** ^1^ Department of Pediatric Endocrinology National Diabetes and Endocrine Center (NDEC) Royal Hospital Muscat Oman moh.gov.om; ^2^ Department of Pediatrics Ministry of Health Muscat Oman moh.gov.om

**Keywords:** case report, FAM20C, hypophosphatemic rickets, osteoporosis, Raine syndrome

## Abstract

**Background:**

Raine syndrome (RS) is a rare autosomal recessive osteosclerotic skeletal dysplasia caused by biallelic pathogenic variants in the FAM20C gene. While the classical phenotype is typically lethal in the neonatal period, an increasing number of nonlethal cases have been recognized. However, long‐term clinical follow‐up data remain extremely limited.

**Case Presentation:**

We report the long‐term follow‐up of a twelve‐year‐old Omani female with a clinical phenotype consistent with nonlethal RS, born to consanguineous parents. Early manifestations included natal tooth, congenital glaucoma, respiratory distress, and extensive intracranial calcifications. During childhood, she progressively developed hypophosphatemic rickets, severe dental abnormalities, growth impairment, skeletal deformities, osteoporosis, and recurrent airway disease. Whole‐exome sequencing identified a homozygous FAM20C variant classified by the reporting laboratory as a variant of uncertain significance (VUS, Class 3). The patient demonstrated clinical and densitometric improvement during bisphosphonate therapy, with reduction in bone pain and improved mobility. A younger sibling with a highly similar clinical phenotype, but who was not genetically tested, exhibited more severe craniofacial and airway involvement, highlighting marked intrafamilial phenotypic variability.

**Conclusions:**

This report provides one of the longest longitudinal follow‐ups of nonlethal RS currently described in the literature. It further expands the phenotypic spectrum of FAM20C‐related disease and offers additional insight into long‐term skeletal complications and therapeutic response. Early recognition and multidisciplinary long‐term follow‐up are essential to optimize outcomes in affected individuals.

## 1. Introduction

Raine syndrome (RS; OMIM #259775) is a rare autosomal recessive disorder first described by Raine et al. in 1989 [1]. The disorder is caused by biallelic pathogenic variants in the ^∗^FAM20C^∗^ gene [2], which encodes a Golgi‐associated kinase involved in biomineralization and phosphate homeostasis [3]. The classical phenotype is characterized by generalized osteosclerosis, craniofacial dysmorphism, intracranial calcifications, severe respiratory compromise, and high neonatal mortality [1, 2]. Since the identification of ^∗^FAM20C^∗^ variants as the underlying molecular defect, the recognized phenotypic spectrum of RS has expanded considerably [4, 5].An increasing number of nonlethal phenotypes have been reported, demonstrating survival into childhood and adulthood [4–6]. Affected individuals may develop progressive skeletal abnormalities, hypophosphatemic rickets, growth impairment, and severe dental complications [5–7]. Considerable intrafamilial phenotypic variability has also been documented [5]. Nevertheless, the natural history of nonlethal RS remains incompletely characterized because of the rarity of the condition and the limited availability of long‐term longitudinal follow‐up data [7].Here, we report a prolonged longitudinal follow‐up of an Omani child with a clinical phenotype consistent with nonlethal RS and a homozygous ^∗^FAM20C^∗^ variant of uncertain significance, highlighting evolving skeletal and extraskeletal manifestations, intrafamilial phenotypic variability, and clinical outcomes during bisphosphonate therapy.

## 2. Case Presentation

### 2.1. Patient Information and Clinical Findings

The index patient is a twelve‐year‐old Omani female born at term to consanguineous parents following an uneventful pregnancy. Birth weight was 2.8 kg. Soon after birth, she was noted to have multiple dysmorphic and craniofacial abnormalities including proptosis, megalocornea, and a natal tooth that was removed during the neonatal period. She also developed congenital glaucoma requiring ophthalmologic intervention.

During the neonatal period, the patient experienced respiratory distress with suspected upper airway obstruction. Choanal atresia was excluded after otolaryngologic assessment. Brain computed tomography (CT) and magnetic resonance imaging (MRI) (Figure 1) demonstrated extensive bilateral intracranial calcifications involving the centrum semiovale and basal ganglia. Congenital infection was initially suspected; however, extensive infectious evaluation and serologic investigations were negative.

**FIGURE 1:**
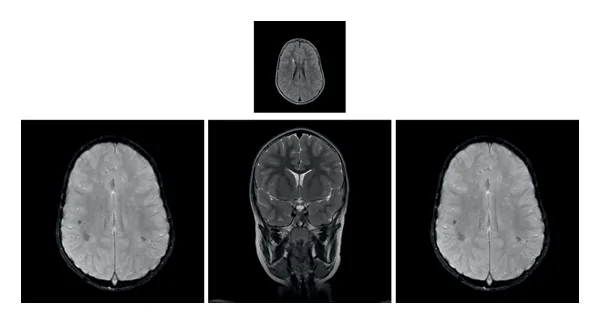
Brain MRI demonstrating extensive intracranial calcifications involving the centrum semiovale and basal ganglia.

At approximately 3 months of age, she developed hepatomegaly associated with transient hypercholesterolemia, hypocalciuric hypercalcemia, and normal liver enzymes. Liver biopsy was nondiagnostic, and these abnormalities resolved spontaneously over time without specific intervention.

At 9 months of age, she developed clinical and biochemical evidence of hypophosphatemic rickets with progressive skeletal deformities. Biochemical investigations demonstrated persistent hypophosphatemia and elevated alkaline phosphatase levels. Treatment with divided oral phosphate supplementation and alfacalcidol was initiated with regular biochemical monitoring. Gross motor development was initially delayed; however, she later achieved independent ambulation and age‐appropriate developmental milestones.

### 2.2. Diagnostic Assessment

Because of persistent hypophosphatemia and skeletal abnormalities, targeted genetic testing for X‐linked hypophosphatemic rickets was initially performed at 4 years of age and was negative. Subsequent whole‐exome sequencing identified a homozygous FAM20C variant, NM_020223.3:c.1646G > A, p.(Arg549Gln) (rs893467130), classified by the reporting laboratory as a missense variant of uncertain significance (VUS, Class 3). Therefore, the molecular finding does not independently confirm RS; the diagnosis was based on the patient’s characteristic clinical and radiological features. Genetic counseling was subsequently provided to the family.

The diagnosis was challenging because the early manifestations involved several organ systems and evolved over time. Extensive intracranial calcifications initially raised concern for congenital infection, but infectious and serological investigations were negative. Persistent hypophosphatemia and rickets subsequently prompted evaluation for hereditary hypophosphatemic disorders; targeted testing for X‐linked hypophosphatemia at 4 years of age was negative. Whole‐exome sequencing at 6 years of age identified a homozygous FAM20C variant, NM_020223.3:c.1646G > A, p.(Arg549Gln), classified by the reporting laboratory as a variant of uncertain significance (VUS, Class 3). Accordingly, this result should not be considered molecular confirmation. The combination of craniofacial abnormalities, intracranial calcifications, severe dental disease, hypophosphatemic rickets, and progressive skeletal manifestations supported the clinical diagnosis of RS. Genetic counseling was provided to the family. A summary of the patient’s clinical course, key diagnostic investigations, and therapeutic interventions from birth to the most recent follow‐up is presented in Table 1.

**TABLE 1:** Clinical course and timeline of disease progression, diagnostic evaluation, and management.

Age/period	Clinical findings/events	Investigation and management
Birth/neonatal period	Term birth; birth weight 2.8 kg. Natal tooth, proptosis, megalocornea, congenital glaucoma, and respiratory distress/upper‐airway symptoms.	Natal tooth removed; ophthalmologic intervention for glaucoma. CT/MRI showed extensive intracranial calcifications. Congenital infection evaluation was negative.
∼3 months	Hepatomegaly with transient hypercholesterolemia and calcium abnormality.	Liver evaluation including biopsy was nondiagnostic; abnormalities subsequently resolved without specific therapy.
9 months	Clinical, biochemical, and radiological hypophosphatemic rickets with progressive skeletal deformities.	Oral phosphate in divided doses and alfacalcidol initiated with biochemical monitoring.
4 years	Persistent hypophosphatemia and skeletal abnormalities.	Targeted genetic testing for X‐linked hypophosphatemia was negative.
6 years	Multisystem phenotype remained unexplained by XLH testing.	Whole‐exome sequencing identified a homozygous FAM20C variant classified as a variant of uncertain significance (VUS, Class 3); clinical diagnosis was based on phenotype.
Childhood	Progressive dental disease, short stature, genu valgum, thoracic scoliosis, limb‐length discrepancy, recurrent airway disease, and chronic musculoskeletal pain.	Ongoing endocrine, dental, ENT, orthopedic, and multidisciplinary follow‐up; phosphate and alfacalcidol continued.
9 years	Worsening back pain, functional limitation, and severe osteoporosis; lumbar spine BMD Z‐score −4.0.	Pamidronate started: 0.5 mg/kg first dose, then 1 mg/kg every 3 months.
October 2020–September 2023	Reduced back pain and improved mobility during treatment; BMD improved to Z‐score −2.9.	Pamidronate continued for approximately 3 years with hydration and clinical/biochemical monitoring.
After September 2023	Mild musculoskeletal pain recurred; dental abscess frequency increased.	Pamidronate discontinued because of limited evidence for prolonged therapy; multidisciplinary surveillance continued.
12 years/current follow‐up	Ongoing skeletal, dental, metabolic, airway, and psychosocial manifestations; pubertal progression without menarche at last assessment.	Long‐term multidisciplinary follow‐up continues.

### 2.3. Clinical Timeline

#### 2.3.1. Long‐Term Follow‐Up and Outcomes

With increasing age, the patient developed progressive multisystem manifestations. Dental complications became particularly prominent, including recurrent gingival abscesses, severe gingival hypertrophy, delayed and abnormal tooth eruption, early tooth loss, high‐arched palate, and severe hypodontia affecting the permanent dentition. She required repeated dental procedures and long‐term specialist dental follow‐up.

Skeletal complications also progressed over time. The patient developed short stature, genu valgum deformity, thoracic scoliosis, limb length discrepancy, chronic musculoskeletal pain, and reduced physical endurance affecting daily activities and mobility. Recurrent adenoid hypertrophy necessitated adenoidectomy, followed by regrowth of adenoidal tissue.

Serial biochemical investigations (Table 2) demonstrated persistent low‐to‐low‐normal serum phosphate levels with intermittently reduced tubular phosphate reabsorption (TmP/GFR). Renal function remained normal throughout follow‐up, and renal ultrasonography showed no evidence of nephrocalcinosis. Abdominal ultrasonography demonstrated persistent hepatomegaly with coarse hepatic echotexture.

**TABLE 2:** Summary of longitudinal biochemical findings.

Parameter	Infancy	Childhood	Latest follow‐up	Normal range
Serum phosphate	0.78	0.97	0.93	(1–1.95) mmol/L
Serum calcium	2.75	2.34	2.16	(1.9–2.7) mmol/L
ALP	800	614	285	(90–210) [IU]/L
PTH	8.7	5.6	8.1	(2–9) pmol/L
Vitamin D	84	113	86	(50–250) nmol/L
TmP/GFR	0.97	0.81	1.05	(1.15–2.4) mmol/L
Renal function (urea, creatinine, and other electrolytes)	Normal	Normal	Normal	

At 9 years of age, worsening back pain and significant functional limitation prompted further skeletal assessment. Bone mineral density evaluation revealed severe osteoporosis with a lumbar spine Z‐score of −4. Skeletal survey showed findings consistent with metabolic bone disease without evidence of generalized osteosclerosis (Figure 2).

**FIGURE 2:**
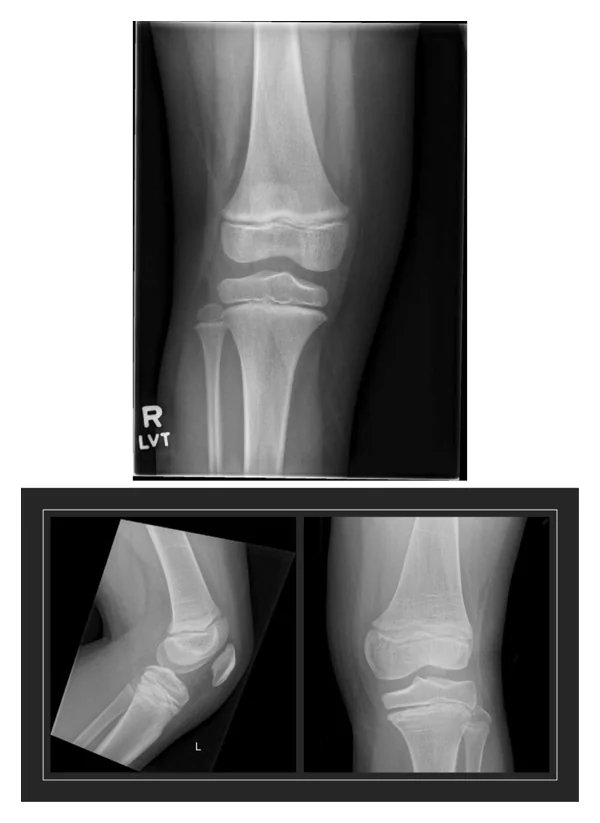
Skeletal survey at 9 years of age showing the absence of generalized osteosclerosis and features consistent with metabolic bone disease.

#### 2.3.2. Therapeutic Intervention and Treatment Response

Given the severity of osteoporosis and persistent bone pain, intravenous pamidronate was initiated at 9 years of age. The first dose was 0.5 mg/kg, followed by 1 mg/kg every 3 months. Treatment was administered from October 2020 to September 2023 (approximately 3 years) in day care with hydration, symptomatic analgesia as required, and biochemical and clinical monitoring. The patient demonstrated marked clinical improvement, with substantial reduction in back pain and improved mobility. Repeat bone mineral density assessment showed improvement in lumbar spine Z‐score from −4.0 to −2.9. No significant treatment‐related adverse events were documented in the available records.

Renal function remained stable, and no nephrocalcinosis was detected during therapy. Dental disease was clinically stable during treatment. Pamidronate was discontinued in September 2023 because of the limited evidence supporting prolonged bisphosphonate therapy in RS. Following cessation, mild musculoskeletal pain recurred and the frequency of dental abscesses increased. These observations are clinically relevant but cannot establish a causal treatment effect in a single patient.

Following cessation of therapy, the patient reported recurrence of mild musculoskeletal pain and increased frequency of dental abscesses. Psychological symptoms including anxiety and low mood emerged during adolescence and were addressed through child psychology services. Formal cognitive assessment demonstrated normal intellectual functioning. At 12 years of age, she had not yet attained menarche but demonstrated ongoing pubertal progression.

#### 2.3.3. Affected Sibling and Intrafamilial Variability

A younger brother developed a highly similar clinical phenotype consistent with RS but was not genetically tested. In addition to features shared with the index patient, he exhibited a more severe phenotype characterized by choanal atresia requiring tracheostomy, craniosynostosis with premature coronal suture fusion requiring neurosurgical intervention, significant proptosis, and extensive intracranial calcifications. The clinical similarity between the siblings, together with the characteristic clinical findings in the index patient and parental consanguinity, supports the clinical diagnosis in the brother while acknowledging the absence of molecular confirmation. This intrafamilial variability highlights the broad phenotypic spectrum of FAM20C‐related disease.

## 3. Discussion

Nonlethal Raine syndrome is increasingly recognized as a clinical phenotype characterized by substantial variability and evolving multisystem manifestations, including skeletal, dental, metabolic, and craniofacial abnormalities [4–6].

Hypophosphatemic rickets is an important clinical manifestation of non‐lethal Raine syndrome, reflecting the essential role of FAM20C in phosphate homeostasis and skeletal mineralization [3, 7]. Similar to previously reported cases, our patient demonstrated persistent hypophosphatemia associated with progressive skeletal disease. Interestingly, generalized osteosclerosis was absent during later childhood, supporting previous observations that osteosclerosis may diminish or be absent in nonlethal phenotypes.

The severe osteoporosis observed in our patient and the favorable response to bisphosphonate therapy represent clinically important observations. Currently, there are very limited published data regarding antiresorptive therapy in RS. The substantial reduction in bone pain and improvement in bone mineral density observed in this case suggest that bisphosphonate therapy may have a therapeutic role in selected patients with severe skeletal involvement.

The marked phenotypic variability observed between the index patient with a clinical diagnosis and her clinically affected, untested younger brother further illustrates the broad clinical variability within families affected by RS. Because the brother did not undergo molecular testing, genotype‐based comparisons between the siblings cannot be made. Recognition of such clinical variability remains important for counseling families and anticipating long‐term complications.

### 3.1. Limitations

This report describes a single patient with a clinical diagnosis of RS and a homozygous FAM20C variant of uncertain significance; it, therefore, cannot establish variant pathogenicity or generalize treatment efficacy. In particular, the observed improvement in pain, mobility, and bone mineral density during pamidronate therapy should be interpreted cautiously because there was no comparator and the natural history of nonlethal RS is incompletely defined. In addition, the clinically affected younger sibling did not undergo molecular testing, limiting genotype–phenotype comparison within the family. Larger longitudinal cohorts and further genetic interpretation are needed to define the natural history and evaluate the safety and effectiveness of skeletal‐directed therapies in RS.

### 3.2. Patient/Parent Perspective

A formal written patient or parent narrative was not prospectively collected. During routine follow‐up, however, the family reported that chronic musculoskeletal pain, mobility limitation, recurrent dental problems, and repeated specialist visits affected the patient’s daily activities. They observed improvement in back pain and mobility during pamidronate treatment. The family agreed to publication of the case to contribute to clinical understanding of this rare disorder.

## 4. Conclusions

Nonlethal RS represents a rare but evolving multisystem disorder associated with significant skeletal, dental, and metabolic morbidity extending into childhood and adolescence. This report provides a detailed long‐term follow‐up of a patient with a clinical phenotype consistent with RS and a homozygous FAM20C variant classified by the reporting laboratory as a variant of uncertain significance. The genetic finding should not be considered molecular confirmation. Long‐term multidisciplinary surveillance remains essential for early recognition of complications and optimization of patient outcomes.

## Funding

This work received no specific grant from any funding agency in the public, commercial, or not‐for‐profit sectors.

## Consent

Written informed consent was obtained from the patient’s parent(s)/legal guardian(s) for publication of the patient’s clinical information and relevant clinical images. Patient confidentiality and anonymity have been maintained. Formal ethics approval for this case report was obtained from Royal Hospital (approval/reference number: MOH/CSR/CR/2628; approval date: 15 July 2026). The report was prepared in accordance with applicable institutional requirements and the principles of the Declaration of Helsinki.

## Conflicts of Interest

The authors declare no conflicts of interest.

## Data Availability

The clinical information supporting this case report is included in the article. Additional patient‐level information is not publicly available because of patient confidentiality and privacy considerations. Further requests for information should be directed to the corresponding author and will be considered subject to appropriate ethical and confidentiality requirements.
